# Prevalence, injury-, and non-injury-related factors associated with anxiety and depression in polytrauma patients – A retrospective 20 year follow-up study

**DOI:** 10.1371/journal.pone.0232678

**Published:** 2020-05-04

**Authors:** Sascha Halvachizadeh, Henrik Teuber, Till Berk, Florin Allemann, Roland von Känel, Boris Zelle, Paolo Cinelli, Hans-Christoph Pape, Roman Pfeifer

**Affiliations:** 1 Department of Trauma, UniversitätsSpital Zürich, Zürich, Switzerland; 2 Harald Tscherne Laboratory, Department of Trauma, University Zurich, University Hospital Zurich, Zurich, Switzerland; 3 Department of Consultation-Liaison Psychiatry and Psychosomatic Medicine, University Hospital Zurich, University of Zurich, Zurich, Switzerland; 4 University of Texas Health Science Centre at San Antonio, San Antonio, TX, United States of America; Monash University, AUSTRALIA

## Abstract

**Introduction:**

Survival rate after polytrauma increased over the past decades resulting in an increase of long-term complaints. These include physical and psychological impairments. The aim of this study was to describe the prevalence and risk factors for developing depression and anxiety more than twenty years after polytrauma.

**Methods:**

We contacted patients who were treated due to a polytrauma between 1973 and 1990 at one level 1 trauma center after more than 20 years. These patients received a self-administered questionnaire, to assess symptoms of depression and anxiety. Analysis based on multivariable logistic regression models include injury severity and non-injury related factors to determine risk factors associated with the development of depression and anxiety.

**Results:**

Patients included in this study (n = 337) had a mean ISS of 20.3 (4 to 50) points. In total, 173 (51.3%) showed psychiatric sequelae (depression n = 163, 48.2%; anxiety n = 14, 4.1%). Injury severity was not associated with the development of depression or anxiety. However, the patients, who required psychiatric therapy prior to the injury had higher risk of developing psychiatric symptoms (OR 1.3, 95%CI 1.1 to 1.8, p = 0.018) as did patients who suffered from additional psychiatric insults after the injury (OR 1.4, 95%CI 1.2 to 2.0, p = 0.049).

**Conclusion:**

More than half of polytrauma patients developed psychiatric sequelae. Risk factors include mainly non-injury related factors such as psychiatric comorbidities and additional psychiatric insults after the injury.

## Introduction

According to the World Health Organization (WHO) injuries are amongst the leading cause of death for children and young adults aged 5–29 years [1]. Survival rates after polytrauma have increased due to improved and standardized treatment strategies [2, 3]. The rate of in-hospital complications of polytraumatised patients decreased substantially after introduction of new fluid resuscitation and transfusion protocols [4]. The survival after otherwise lethal injuries based on advancements in acute care strategies [4, 5]. This development has led to an increase of long-term complaints including functional impairments [6] or psychiatric sequelae [7]. In 2016, O’Donnell et al [8] found that 28% of patients met criteria for at least one psychiatric complication after 72 months after severe injury. Further, they described depressive episodes to be the most prevalent psychiatric complication after severe trauma [8]. A high rate of studies on psychiatric sequelae after trauma focus on military conflict setting [9]. However, a growing body of literature investigates psychiatric sequelae after civilian injuries [10, 11]. Long-term studies focusing on long-term psychiatric sequelae after injuries are increasing in number [8, 12–14]. Yet, studies still are inconsistent on risk factors that promote psychiatric sequelae after trauma. Some investigations on psychiatric sequelae after trauma discuss injury-related factors and injury severity as potential risk factors for anxiety [15] other studies describe non-injury related factors, including history of psychiatric illness, frailty [16], alcohol abuse, and female gender, to be associated with anxiety or depression [17].

Yet, no study investigated the prevalence and risk factors for the development of anxiety and depression after polytrauma more than 20 years after injury. This study aimed to answer the following questions

- What is the prevalence of anxiety and depression more than 20 years after polytrauma?
- Is there an association of specific injured body region or severity of injury with the development of anxiety or depression?
- What non-injury related factors, since the trauma, are associated with the development of anxiety or depression?

## Methods

This study was approved by the institutional review board of the Hannover Medical School (Ethical Committee Trial ID-Number 2325-200/03/22).

### Long term follow up database: Baseline data

The initial database included selected patients that were treated due to a polytrauma at one level 1 trauma centre. These data were collected between January 1^st^, 1973 and December 31^st^, 1990 at a as described previously [18]. The initial selection criteria cannot be reproduced, however, the collected variables included: ISS, Abbreviated Injury Scale (AIS) for AIS specific body regions [19], patients’ demographic (age at injury, gender, marital status), total length of stay, and length of stay at the intensive care unit (ICU). The treating trauma team collected the measurements, including AIS, during the development of the long-term database under the supervision of our co-authors (BZ and HCP). The total number of patients included in this database was 637. The AIS was re-calculated when patients were contacted and the AIS version 2005 were utilized to update the provided AIS. ISS and AIS represent injury severity and injury distribution based on anatomic injuries. ISS is calculated based on the three highest AIS per body region according the following formula [20]: AIS_1_^2^ + AIS_2_^2^ + AIS_3_^2^

### Study population: The recruitment process

Patients were contacted between 2007 and 2011. The first step of contacting patients included a phone call for confirmation and update of their address and the readiness to participate in the study. In the second step, the patients received the questionnaire and an informed consent form by mail. The third step involved the return of the signed form and the questionnaire that both served as written informed consent. The last included questionnaire was returned in 2011. Inclusion criteria were treatment due to a polytrauma at one Level 1 trauma centre between 1973 and 1990. Polytrauma was defined by an injury severity score (ISS) of 16 points or higher, and multiple injuries from which one, or the combination of injuries are life threatening, defined by an AIS cut-off of 4 or higher [21]. Further, only patients that could be contacted and returned a questionnaire with a written informed consent were included in this study. At baseline, exclusion criteria included genetic disorders or oncologic disease; at follow-up, incomplete data, incomplete questionnaire, or illegible questionnaire led to exclusion.

### Questionnaire and follow up data

The self-administered questionnaire consisted of 118 questions. These inquire about psychiatric symptoms, quality of life, work status, and current or recent psychotherapeutic treatment or rehabilitation. A German version of the 14-item self-reported Hospital Anxiety and Depression Scale (HADS) [22] assessed symptoms of depression and anxiety. Each item was rated on a 4-point Likert scale (range from 0 = “mostly” to 3 = “not at all”). The seven item subscales yield a score of 0 to 21 points each. The level of anxiety and depressive symptoms is interpreted as normal (0–7 points), mild (8–10 points), moderate (11–14 points) or severe (15–21 points). Diagnosed psychiatric conditions requiring regular psychiatric treatment for more than one month, including medication or psychotherapy defined psychiatric treatment. Further, the questionnaire assessed additional psychiatric insults after the injury: These included the experience of secondary severe injury, fire in the patients’ residence that required assistance from the fire department, explosions, life threatening diseases, physical, or sexual assault. The research team developed these questions, since no validated screening tools exist, that evaluates the aforementioned topics. The questionnaire further included the following questions: Were you in need of psychiatric therapy prior or after trauma? Did you require inpatient psychiatric therapy? Are you retirement, or unemployment due to accident? These questions were summarized as non-injury related factors.

### Statistics

Continuous data are presented with mean (±Standard Deviation, SD), categorical variables with numbers and percentages. Group comparisons (two parties) on continuous variables were performed using student’s t-test; group comparisons (two parties) on categorical or binary variables were performed using Pearson chi-square test. Odds ratios were calculated based on Multivariable logistic regression model. Outcome variable included depression, anxiety, or both (anxiety and depression) as measured by the score obtained from the questionnaire (≥ moderate, 11–21 points), the comparators are the patients without these psychiatric symptoms. Model coefficient included injury severity (ISS and AIS), and non-injury related factors (gender, age at injury, additional psychiatric insults, psychiatric therapy prior or after the injury, inpatient psychiatric treatment, retirement, unemployment due to the injury, length of stay and length of ICU stay). These variables have known clinical influence or approached significance (p ≤ 0.2). Thus the analysis were multivariable on fixed effect. The variables in the model were assumed to be independent. Statistical analysis was performed using R (R Core Team (2018). R: A language and environment for statistical computing. R Foundation for Statistical Computing, Vienna, Austria. URL: https://www.R-project.org/).

## Results

### Patients

**Fig 1:**
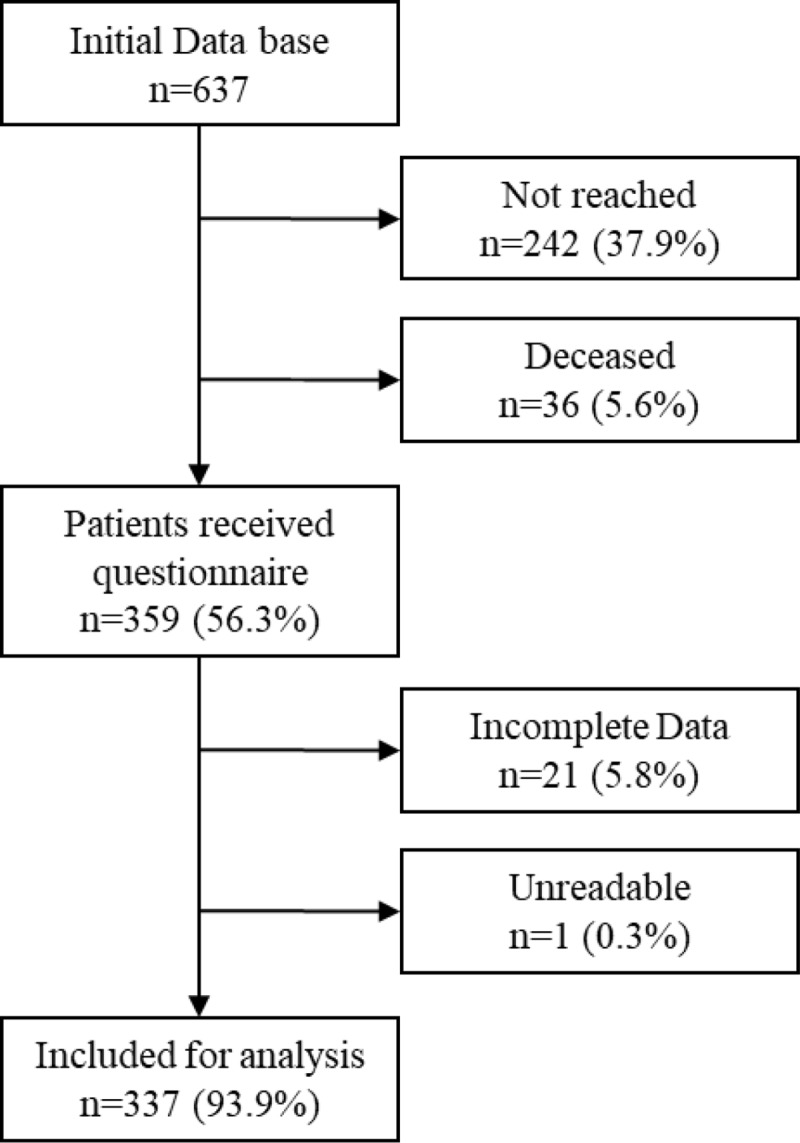
Flow chart of inclusion process of study population. The initial database included XXX patients. The exhaustive process to contact patients included contacting regional administration offices (up to three different offices) to follow patients who moved.

After using the recruitment process [23] with multiple steps to identify and contact patients that had moved, 242 (37.9%) patients could not be reached and 36 (5.6%) deceased according to our records. In total, 359 (56.3%) patients were both contacted by phone and received a questionnaire. Out of the 359 patients, 21 (5.8%) had incomplete data and one (0.3%) patient returned an illegible questionnaire. Thus 337 (93.9%) out of eligible 359 patients met the inclusion criteria. According the STROBE guidelines a participant flow chart is displayed in Fig 1. A comparative analysis of the non-contributor (excluding patients who deceased) to this study revealed no statistical significant differences in age, gender, ISS, or length of stay. Patients were 25.4 (±11.7) years old at the time of the injury; their mean age at the time of questionnaire was 54.1 (±12.3) years. Patients’ demographics are displayed in Table 1. Mean follow-up time was 28.5 (±6.8) years. In total 71 (21.1%) patients were in regular psychiatric treatment for at least one month prior to the injury. The demographic data, as well as the injury severity, injury distribution, or length of hospitalization of patients in psychiatric treatment prior to the injury was comparable to the data of patients without prior psychiatric treatment. A significantly higher percent of patients who were unemployed since the trauma (at the most recent follow-up) had psychiatric treatment prior to the injury (31.4% versus 17.3%, p = 0.04).

**Table 1:** Descriptive summary of demographics of study population.

	Studypopulation	Non-contributors
n	337	264
Age at injury [years], mean (SD)	25.4 (11.7)	29.5 (14.3)
Gender female n (%)	76 (25.3)	61 (23.1)
married n (%)	107 (35.8)	93 (35.2)
Length of Stay [days], mean (SD)	29.8 (23.4)	25.4 (19.7)
Length of Stay ICU [days], mean (SD)	6.9 (10.3)	8.9 (12.3)
ISS [points], mean (SD)	20.3 (9.3)	20.8 (9.8)
Unemployed since injury n (%)	49 (20.9)	
Patients suffered additional psychiatric insult after injury n (%)	88 (26.0)	

n = Number

ICU = Intensive Care Unit

ISS = Injury severity score

### Prevalence of depression and anxiety

In total, 163 (48.2%) patients had 11 to 21 points on the HADS scale for depression; 14 (4.1%) had 11 to 21 points on the HADS for anxiety. These patients had per definition symptoms indicative of clinical depression, or anxiety. Four patients (1.2%) had symptoms of both depression and anxiety. The demographics of patients suffering from clinically elevated symptoms of depression or anxiety are summarized in Table 2.

**Table 2:** Comparison of demographics of patients stratified according to psychiatric sequelae after injury. Stratification performed according to total score on HADS. Exploratory p-values based on ANOVA and chi-squared test.

	Healthy	Depression	Anxiety	Anxiety and Depression
Number of patients (%)	157 (46.4%)	163 (48.2%)	14 (4.1%)	4 (1.2%)
Age at injury [years], mean (SD)	25.3 (12.5)	25.35 (12.5)	26.38 (13.0)	19.75 (4.6)
Gender female n (%)	31 (24.2)	36 (25.9)	1 (12.5)	3 (75.0)
Length of Stay [days], mean (SD)	27.5 (22.2)	31.65 (24.5)	13.00 (7.9)	32.25 (14.9)
Length of Stay ICU [days], mean (SD)	12.9 (10.8)	12.30 (12.0)	12.33 (10.5)	8.75 (5.6)
ISS [points], mean (SD)	20.5 (9.5)	19.49 (8.9)	23.50 (7.1)	28.00 (17.6)
Additional psychiatric insult after injury n (%)	41 (29.3)	35 (22.3)	5 (50.0)	1 (25.0)
Psychiatric therapy after injury n (%)	3 (2.2)	2 (1.3)	0 (0.0)	1 (25.0)
Need of psychiatric therapy prior the injury n (%)	42 (30.4)	13 (8.4)	7 (70.0)	4 (100.0)
Unemployment since the accident n (%)	20 (21.3)	18 (15.8)	5 (55.6)	2 (50.0)

n = number

Healthy includes patients without elevated symptoms (HADS points 10 or less)

Depression and anxiety includes patients with clinical relevant symptoms as indicated by HADS with 11 or more points

ICU = Intensive Care Unit

ISS = Injury Severity Score

Further, 82 (24.3%) patients were categorized as moderate depressive (8–10 points), and 92 (27.3%) patients had no depressive symptoms (0–7 points). In comparison 44 (13.1%) patients were categorized as moderate anxiety and 279 (82.8%) of patients had no symptoms of anxiety.

### Depression and anxiety

Injury related factors that are associated with symptoms of depression or anxiety are summarized in Table 3. The severity of injuries were not associated with the development of depression, anxiety or both.

**Table 3:** Association of injury-related and non-injury-related factors with the development of psychiatric sequelae. The Reference are patients, that did not suffer from symptoms of depression or anxiety.

		Depression			Anxiety			Anxiety and Depression		
		OR	95%CI	p-value	OR	95%CI	p-value	OR	95%CI	p-value
Injury severity	ISS	1.0	1.0–1.1	0.457	1.1	0.9–1.2	0.578	1.3	1.0–1.7	0.071
	AIS Head	0.9	0.7–1.1	0.255	1.1	0.5–2.5	0.778	0.7	0.2–2.3	0.542
	AIS Face	0.9	0.6–1.2	0.332	0.7	0.3–1.8	0.485	0.4	0.1–2.2	0.272
	AIS Thoracic	0.9	0.7–1.1	0.454	1.0	0.5–1.9	0.931	0.4	0.1–1.3	0.122
	AIS Abdomen	0.9	0.7–1.2	0.549	0.8	0.4–1.6	0.483	1.4	0.7–3.0	0.33
	AIS Spine	0.8	0.6–1.1	0.119	0.8	0.3–2.2	0.61	NA	NA	NA
	AIS Upper Extremity	0.9	0.8–1.2	0.559	1.6	0.8–3.3	0.195	1.0	0.4–2.6	0.988
	AIS Lower Extremity	1.1	0.9–1.4	0.429	1.0	0.5–2.2	0.9	1.3	0.3–6.3	0.779
Non-injury related factors	Female Gender	1.4	0.5–3.4	0.153	1.1	0.0–26.3	0.598	NA	NA	NA
	Age at injury	1.0	1.0–1.1	0.163	0.9	0.8–1.1	0.784	NA	NA	NA
	Need of psychiatric therapy prior the injury	1.3	1.1–1.8	0.018	5.7	0.2–17.6	0.064	NA	NA	NA
	Psychiatric therapy after injury	0.4	0.0–4.7	0.987	14.1	0.1–37.1	0.373	NA	NA	NA
	Retired since injury	0.9	0.7–1.3	0.977	2.2	0.5–10.6	0.087	NA	NA	NA
	Unemployed due to injury	1.0	0.4–2.6	0.875	0.6	0.0–78.2	0.075	NA	NA	NA
	Additional psychiatric insult after injury	1.4	1.2–2.0	0.049	16.4	0.2–14.3	0.44	NA	NA	NA
	Length of stay in ICU	1.0	1.0–1.0	1	1.0	0.9–1.2	0.881	NA	NA	NA

n = number

Depression and anxiety includes patients with clinical relevant symptoms as indicated by HADS with 11 or more points

ICU = Intensive Care Unit

ISS = Injury Severity Score

AIS = Abbreviated Injury Scale

Amongst the non-injury related factors, patients in need of psychiatric treatment prior the injury are associated with increased risk of developing depression (OR 1.3, 95%CI 1.1 to 1.8, p = 0.018). Further, the risk of developing symptoms of depression is increased in patients who suffered additional psychiatric insults after the injury (OR 1.4, 95%CI 0.2 to 2.0, p = 0.049).

## Discussion

Psychiatric sequelae are common after injuries and often cause devastating problems for individuals and their families [24, 25]. According to the WHO 3.4% of the world population suffers from depression [26] that accounts for one important factors affecting the Disability Adjusted Life Years [27].

The aim of this study was to assess the long-term psychiatric outcome with a special focus on development of anxiety and depression more than 20 years after polytrauma and to describe risk factors for the development of these psychiatric sequelae. Our main results were as follows:

- More than half of the patients (51.3%) showed symptoms indicative of either clinical depression or anxiety more than 20 years after polytrauma.
- Injury severity was not associated with the development of either depression, anxiety, or both.
- Non-injury related factors associated with depression were additional psychiatric insults, and patients in need of psychiatric treatment prior to the injury.

### Prevalence of psychiatric sequelae after polytrauma

Our group described a rate of PTSD of nearly 3% twenty years after severe trauma, without association to injury severity or distribution [28]. Psychiatric sequelae after polytrauma or severe trauma are most commonly described in military setting. A recently published systematic review on psychiatric comorbidities among older veterans summarized a prevalence of depressive disorder of 33% to 52.3% and a generalized anxiety disorder of 14% to 15% [29]. These rates for depression are comparable to this study (48.2%), however, the percentage of patients with symptoms of anxiety in our cohort was lower (4.1%). One study on civilian polytrauma patients revealed a rate of depression or anxiety of 28% after a mean of 14 months (12 to 18 months) after polytrauma [30]. A similar follow-up period revealed that more than 54% of polytrauma patients show signs of depression and anxiety [31]. Compared to the life time prevalence of major depressive episodes, that have been reported to range from 12.2% [32] to 18% [33], patients suffering major trauma, either in military or civilian, are at increased risk of developing psychiatric sequelae. There is urgent need for screening, education of patients and medical personnel, and appropriate consultation of psychiatric health professionals in the treatment of polytrauma patients.

### Risk factors for developing psychiatric sequelae after polytrauma

The association of injury severity and injury distribution and the development of psychiatric sequelae is inconsistent. Our study revealed that injury severity of the spine is associated with both the development of depression and anxiety. Further, the overall ISS was not associated with the development of psychiatric sequelae. A study on polytrauma patients in the Netherlands also showed a significant association of injuries to the spine and the development of psychiatric sequelae [30]. A growing body of literature investigates the association of traumatic brain injuries and the development of psychiatric sequelae. Similar to our results, it has been shown that the severity of brain injury is associated with the development of psychiatric sequelae [34]. It appears that injury severity of the central nervous system is associated with an increased risk of developing psychiatric sequelae. Polytrauma patients with sequelae brain injuries or spinal cord injuries are at increased risk for developing psychiatric sequelae.

According to this study, additional psychiatric insults and the need of psychiatric treatment are associated with the development of psychiatric sequelae. Psychiatric stressors lead to increased risk of developing psychiatric diseases [35]. This sensitivity to psychiatric sequelae increases the risk of the development of depression, or anxiety after trauma [36]. It appears, that patients who suffered psychiatric insults or are at higher risk of experiencing psychiatric stressors are at high risk of developing psychiatric sequelae after polytrauma.

### Strengths and Limitations

The strength of this study is the long-term follow-up period (more than 20 years) and the homogeneity of treatment strategy of the polytrauma patient. Some might argue that the time as a continuous variable might place an artificial and arbitrary constraint on the analysis. As shown above, the rate of psychiatric sequelae are not substantially different comparing one to two year follow-up to long-term follow up studies. We are aware, that the rate of psychiatric sequelae changes over time, however, we feel confident in our results, since these are comparable to current literature. The evaluation of psychiatric sequelae more than 20 years after trauma is unique in the literature. However, patients from the long-term follow-up data base filled out the questionnaire by themselves, representing a subjective self-evaluation. Limitations of filling out the questionnaire due to age or trauma sequelae might be considered as confounding factors. There is a possibility that some patients who did not consent to participate, or did not return the questionnaire, are unwilling to discuss and relive possible psychiatric situation of their past trauma. This might have caused underreporting of long-term psychiatric sequelae, which cannot be completely be ruled out.

This is the first study that assessed the prevalence and risk factors for developing depression or anxiety more than twenty years after polytrauma in civilians. The prevalence of these psychiatric sequelae exceeds 50%. Patients who suffered injuries of the central nervous system as well as patients who have suffered or are at risk to suffer psychiatric insults (secondary severe trauma, physical or sexual assault, war etc) are at increased risk of developing psychiatric sequelae. The long-term results reported here can serve as an important impulse to raise awareness of psychiatric sequelae after polytrauma, especially in patients at increased risk.

## Conclusion

More than half of the patients that suffered a polytrauma are at risk of suffering depression or anxiety. Special care should be given to polytrauma patients who are prone to psychiatric sequelae based on additional psychiatric insults and who were in need of psychiatric treatment.
